# Microplastics-Induced Eryptosis and Poikilocytosis in Early-Juvenile Nile Tilapia (*Oreochromis niloticus*)

**DOI:** 10.3389/fphys.2021.742922

**Published:** 2021-09-28

**Authors:** Mohamed Hamed, Alaa G. M. Osman, Ahmed E. A. Badrey, Hamdy A. M. Soliman, Alaa El-Din H. Sayed

**Affiliations:** ^1^Department of Zoology, Faculty of Science, Al Azhar University (Assiut Branch), Cairo, Egypt; ^2^Department of Zoology, Faculty of Science, Sohag University, Sohag, Egypt; ^3^Zoology Department, Faculty of Science, Assiut University, Asyut, Egypt

**Keywords:** microplastics, poikilocytosis, apoptosis, tilapia, *Oreochromis niloticus*, erythrocytes

## Abstract

This study aims to assess the impact of microplastics (MPs) on erythrocytes using eryptosis (apoptosis) and an erythron profile (poikilocytosis and nuclear abnormalities), considered to be novel biomarkers in Nile tilapia (*Oreochromis niloticus)*. In this study, four groups of fish were used: The first was the control group. In the second group, 1 mg/L of MPs was introduced to the samples. The third group was exposed to 10 mg/L of MPs. Finally, the fourth group was exposed to 100 mg/L of MPs for 15 days, following 15 days of recovery. The fish treated with MPs experienced an immense rise in the eryptosis percentage, poikilocytosis, and nuclear abnormalities of red blood cells (RBCs) compared with the control group in a concentration-dependent manner. Poikilocytosis of MP-exposed groups included sickle cell shape, schistocyte, elliptocyte, acanthocyte, and other shapes. Nuclear abnormalities of the MPs-exposed groups included micronuclei, binucleated erythrocytes, notched, lobed, blebbed, and hemolyzed nuclei. After the recovery period, a greater percentage of eryptosis, poikilocytotic cells, and nuclear abnormalities in RBCs were still evident in the groups exposed to MPs when crosschecked with the control group. The results show concerning facts regarding the toxicity of MPs in tilapia.

## Introduction

Plastics consist of small monomers polymerized with supplements of additives, such as stabilizers, plasticizers, and pigments (Xu et al., 2019). Approximately, 300 million tons of manufactured plastics are used in industrial processes and food packing each year (PlasticsEurope., 2015). Most of the plastic wastes are discarded in aquatic ecosystems, especially in developing countries (Karbalaei et al., 2018).

There is a relatively high presence of microplastics (MPs) in freshwater bodies adjacent to the highly populated urban areas (Eriksen et al., 2013; Yonkos et al., 2014; Zhao et al., 2014; Lasee et al., 2017). Eriksen et al. (2013) found an approximate average value of MPs (mesh size 333 mm) at 43,000 particles/km^2^ with the highest abundance areas containing over 466,000 particles/km^2^ adjacent to the major cities. Lasee et al. (2017) found that the average MP concentrations in samples obtained from lakes ranged from non-detectable (ND) to 5.51 ± 9.09 mg/L for varying size fractions. Even though it seems that the MP concentrations are directly proportional to the urban population, the research showed relatively high concentrations of MPs in remote freshwater environments with extremely low population density and industrialization (Free et al., 2014; Yonkos et al., 2014; Fischer et al., 2016). It has been hypothesized that the urban water systems are immensely underrepresented in the MP life cycle despite their potential to significantly alter the microbial distributions by availing downstream transport and increased levels of plastic-consuming microbes in these water systems (McCormick et al., 2014).

The toxic effects of MPs on the aquatic organisms are debatable and differ across the species according to the size and type of MPs as well as the presence of heavy metals and pesticides (Rainieri et al., 2018; Wu et al., 2019). In fish, several adverse effects, include diminished predatory prowess, endocrine inhibition, oxidative stress, hepatic stress, and intestinal alterations (Rochman et al., 2013; Ferreira et al., 2016; Pedà et al., 2016; Hamed et al., 2019a, 2020). Although there are many pieces of research on the MPs effects on fish, still, there is not enough information on the effect of MPs on the alterations of blood cells and molecular damage especially on the Nile tilapia (*Oreochromis niloticus*) (Hamed et al., 2019a, 2020). Hematological, biochemical, and antioxidant alterations recorded in the fish after exposure to MPs are attributed to their toxic effects; however, little is known about the damage mechanism inside the cells and tissues (Hamed et al., 2019a, 2020). Understanding the mechanism of cellular damage associated with MP exposure would help develop protective treatments at both the cellular and tissue levels. Blood and its constituents represent the first point of entry for MPs in fish due to the direct contact between the gills and contaminated water (Barboza et al., 2020). Accordingly, the erythrocytes in target organs offer a unique tool for assessing the cytotoxicity in fish (Fazio, 2019; Soliman and Sayed, 2020). Eryptosis and erythron profiles (poikilocytosis and nuclear abnormalities) of fish erythrocytes are valuable biomarkers of the toxicity of different chemicals, pharmaceutical residues, and ultraviolet radiation (Mekkawy et al., 2011; Morina et al., 2012; Sayed et al., 2013, 2015, 2016, 2017; Sayed, 2016; Sula et al., 2020). The internal health conditions of the fish can be classified according to the indices iterating serum biochemical, hematological, and immunological aspects (Edsall, 1999; Luskova et al., 2002). This allows using these alterations to study the mechanisms underlying the hazardous effects caused by pollutants (Luskova et al., 2002). The hematological properties, such as red blood cell (RBC), hematocrit (Ht), and hemoglobin (Hb) are vital indicators for the evaluation of the health status of fish after being exposed to different environmental stresses, bacterial infections, and chemical toxicity (Kim et al., 2019, 2020). MP invades the circulatory system of fish, causing lethal reactions and metabolic disorders, e.g., endocrine disorders, oxidative stress, gene expression, and immune responses (Ma et al., 2020). Fish-absorbed MPs diffuse into the blood system through the cell membrane, causing accumulation in the body. Hence, the hematological aspects can be sensitive indicators of MP exposure in fish (Scanes et al., 2019). Hamed et al. (2019a) stated that the blood properties, e.g., hemogloboin, RBCs, and hematocrit, of *O. niloticus* were altered after being exposed to MP. This phenomenon caused the blood hemodilution after tissue damage and MP toxicity–induced hemolysis.

The Nile tilapia is a prominent fish in aquaculture today. It acts as a valuable experimental model in toxicological studies since it can be easily manipulated and observed under experimental conditions (Soliman, 2017). To the best of our knowledge, this is the first study that aims to examine the cytotoxicity of MPs in tilapia using novel biomarkers, such as eryptosis, poikilocytosis, and nuclear abnormalities of RBCs.

## Materials and Methods

### MPs and Stock Preparation

Microplastics were virgin powder with irregular-shaped particles (more than 90% of MPs and 100 nm in size) purchased from Toxemerge Pty Ltd (Australia). MPs characterization occurred per the procedures described in Hamed et al. (2019a). The stock solution was prepared as per the manufacture protocol, stored at a temperature of 4°C in the dark. The stock solution (2.5 g MP/L) was mixed with purified water (Milli-Q). Then, it was sonicated before each use. A further diluted solution for the test concentrations was prepared from this stock right before the beginning of each experiment.

### Fish Exposure

Early juvenile male tilapia (*n* = 120 fish, weight 4.35 ± 0.067 g, total length 3.28 ± 0.12 cm) was transported into aired tanks from the Aquaponic Unit at Al-Azhar University to the Laboratory of Fish Biology (Faculty of Science, Assiut University, Egypt). The fish (fed a commercial pellet diet with 3% of body weight per day) were acclimatized for 2 weeks in good-quality water within terms of parameters, such as conductivity (260.8 μ M/cm), pH (7.4), dissolved oxygen content (6.9 mg/L), temperature (28.5°C), NH_4_ (<1 mg/L), NO^−3^(<1 mg/L), and NO^−2^ (<1 mg/L), and photoperiod (12:12 h light:dark). The fish were divided into four groups of 30 individuals each (10 per each replicate): a control group and three groups treated with MPs at concentrations of 1, 10, and 100 mg/L for 15 days followed by 15 days of recovery, according to a method described by Katzenberger and Thorpe (2015). During the exposure period, the water was renewed daily (40%) to reduce the impurities from metabolic wastes and MPs were re-dosed daily, while during the recovery period, the water was renewed daily to diminish the impurities from metabolic wastes without the dosing of MPs.

At the end of the experiment, six fish from each group were randomly handpicked and anesthetized using ice to lessen stress (Hamed et al., 2019a). The blood samples taken from the caudal vein for blood smears were used for eryptosis and erythron profiles (to measure poikilocytosis and nuclear abnormalities). The experimental setup and fish handling were approved by the Research Ethics Committee of the Faculty of Science, Assiut University, Assiut, Egypt.

### Erythrocyte Programmed Cell Death (Eryptosis)

Eryptosis was detected following the procedures described by Sayed et al. (2016). In brief, the blood smears were prepared and stained with acridine orange, and RBCs were then observed under a fluorescence microscope (Zeiss Axioplan2) equipped with a digital 3 CCD color video camera (Sony, AVT-Horn, Japan).

### Erythron Profiles (Poikilocytosis and Nuclear Abnormalities of RBCs)

The blood smears were assorted, stained with hematoxylin and eosin, handpicked, classified with codes, randomized, and scored blindly for erythrocyte alterations and nuclear abnormalities. For every group, 10,000 cells (1,000 per slide minimum) were analyzed at 100× magnification for MN and altered RBCs in the varying groups. The criteria of observation for MN counts were previously detailed by Al-Sabti and Metcalfe (1995) and Schmid (1975).

### Statistical Analysis

All data were assessed in SPSS (SPSS 1998, SPSS Inc., Chicago, IL, USA) at *p* < 0.05. Data were subjected to a Shapiro–Wilk test for normality and Levene's test for homogeneity of variances utilizing the one-way ANOVA test. Should there be variance equality, Fisher's least significant difference *post-hoc* test was employed to crosscheck the treated groups against the control group. Should there be variance inequality, Dunnett's *post-hoc* test instead employed to crosscheck the treated groups against the control group.

## Results

### Erythrocyte Programmed Cell Death (Eryptosis)

**Figure 1:**
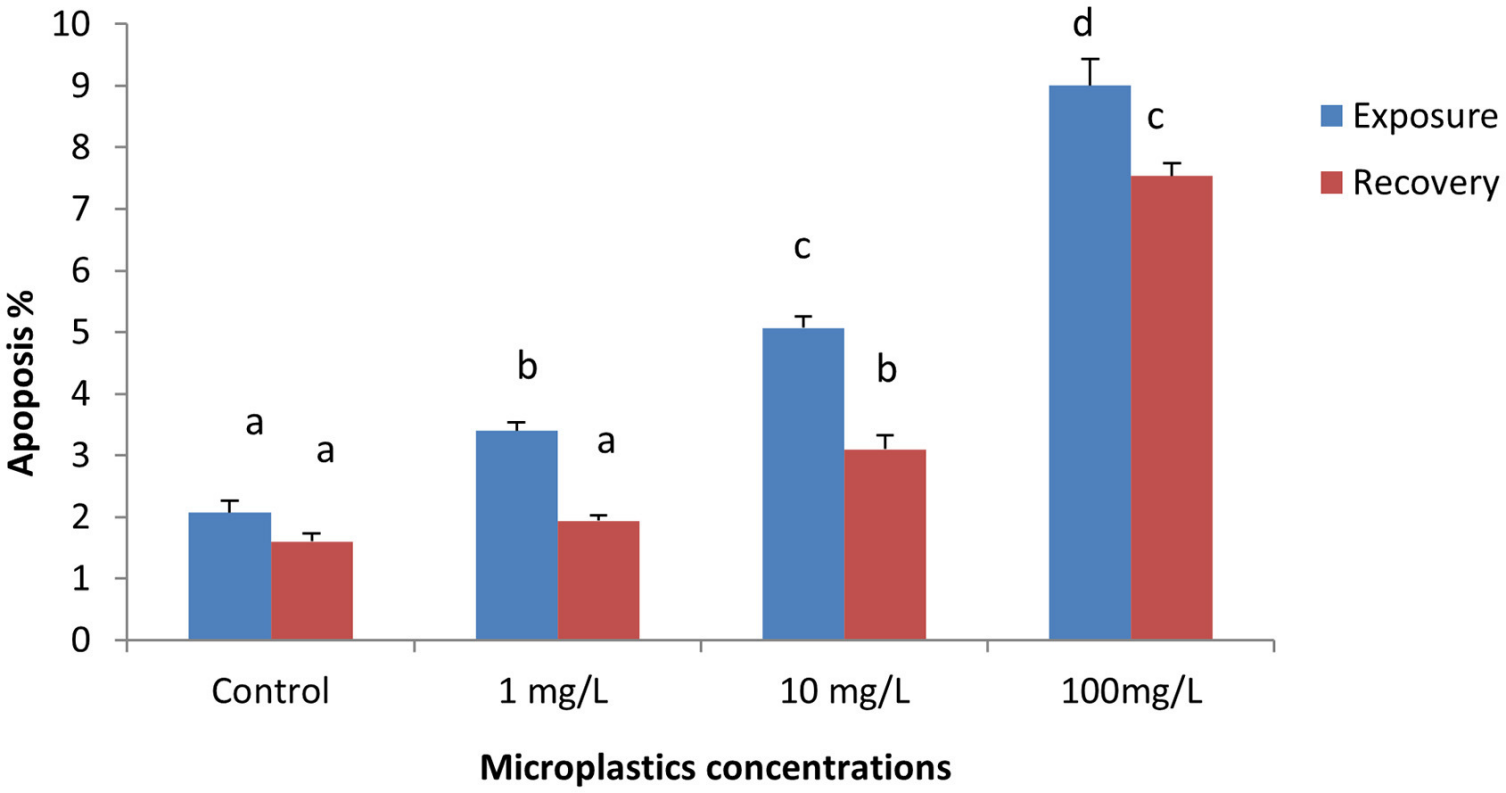
Percentage of apoptotic cells of early-juvenile tilapia (*Oreochromis niloticus*) exposed to microplastics (MPs). Data are presented as mean ± SE. Bars with different superscript letters are significantly different (*P* < 0.05) (*n* = 6).

**Figure 2:**
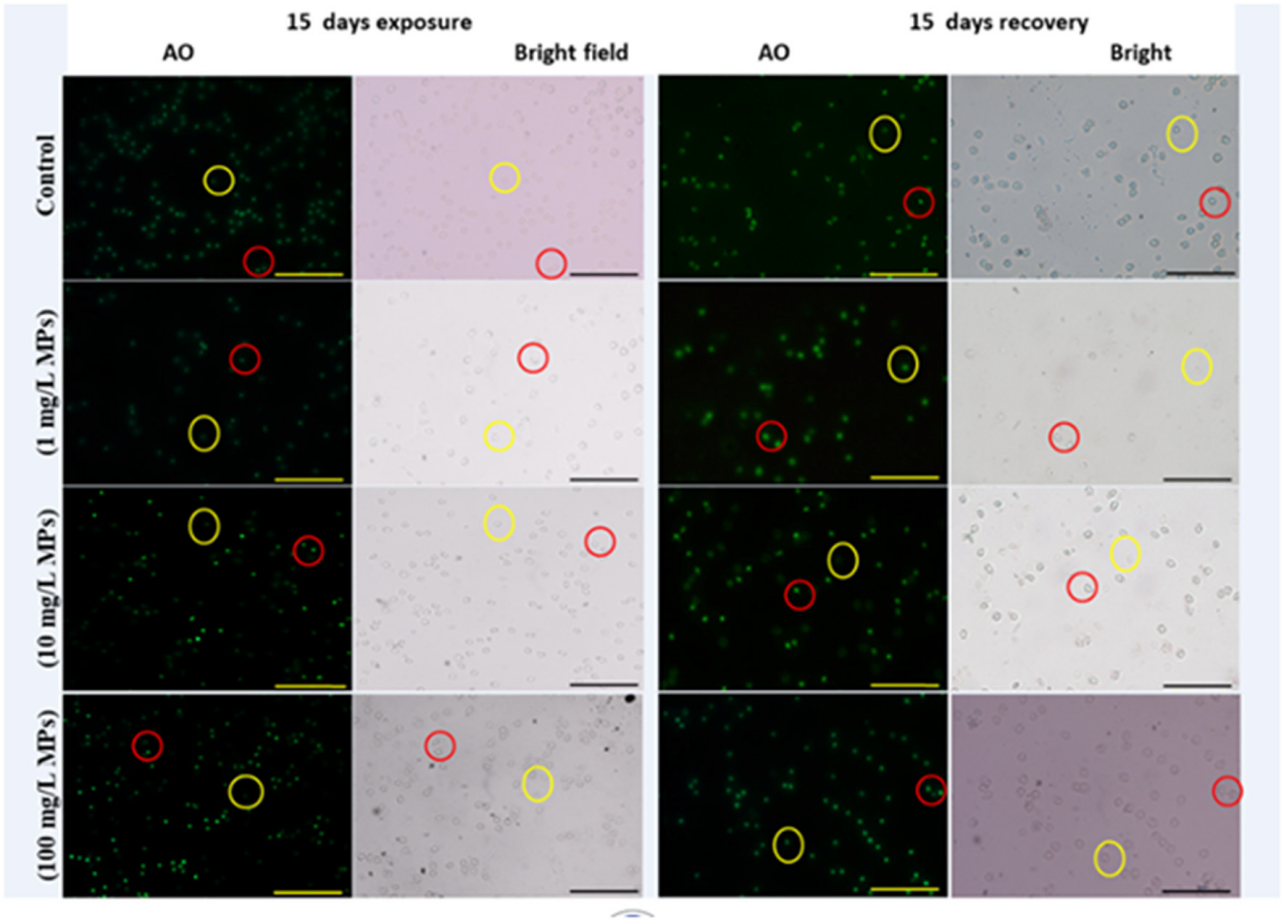
Apoptosis detection in early-juvenile tilapia (*O. niloticus*) after exposure and the recovery to control conditions and MPs at 1, 10, and 100 mg/L. The cells fluorescing light green after staining with acridine orange was considered apoptotic, while normal cells were pale green. Red circles indicate apoptotic cells and yellow circles indicate nonapoptotic cells. (Scale bar = 50 μm).

The percentage of apoptotic cells increased significantly in the MPs-exposed groups compared with the control group in a concentration-dependent manner (Figure 1). RBCs of the control group appeared normal with pale-green nuclei (Figure 2), while those of the MPs-exposed groups appeared apoptotic with luminous light-green nuclei (Figure 2). After the recovery period, a higher percentage of apoptotic cells was observed in the MPs-treated groups compared with the control group in a concentration-dependent manner (Figures 1, 2).

### Erythron Profiles (Poikilocytosis and Nuclear Abnormalities of RBCs)

**Figure 3:**
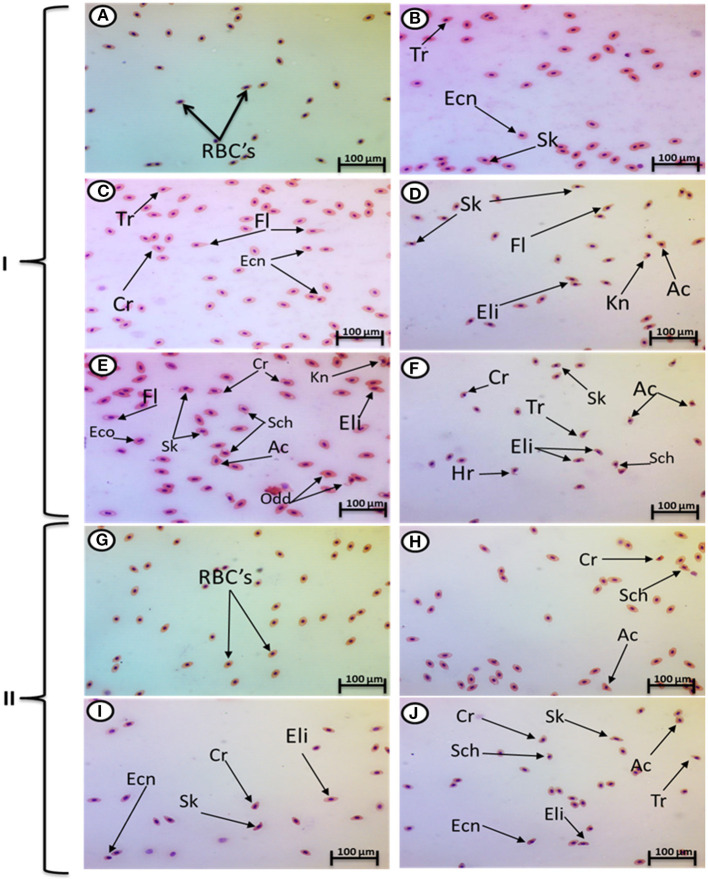
Hematoxylin and eosin-stained blood smears from juvenile *O. niloticus* showing I; MP exposure: normal erythrocytes **(A)**, deformed ones after exposure to MPs at 1 mg/L for 15 days **(B)**, deformed ones after exposure to MPs at 10 mg/L for 15 days **(C,D)**, and deformed ones after exposure to MPs at 100 mg/L for 15 days **(E,F)** and II; recovery period: normal erythrocytes **(G)**, deformed ones after recovery from exposure to MPs at 1 mg/L for 15 days **(H)**, deformed ones after recovery from exposure to MPs at 10 mg/L for 15 days **(I)**, and deformed ones after recovery from exposure to MPs at 100 mg/L for 15 days **(J)**. Tr, tear-drop cell; Cr, crenated cell; Ac, acanthocyte; Ecn, eccentric nucleus; Kn, kidney shape; Hr, heart shape; Eli, elliptocytes; odd, odd shape; FL, filamented; Shc, schistocytic and Sk, sickle cell.

Exposure to 1, 10, and 100 mg/L of MPs was associated with a significant rise in the percentage of poikilocytosis and nuclear abnormalities of RBCs compared with the control group in a concentration-dependent manner (Tables 1, 2). The blood smears of the control group contained normal erythrocytes ellipsoidal in shape with a centrally located ellipsoidal nucleus (Figure 3A). The blood smears of the fish exposed to MPs at 1 mg/L displayed poikilocytosis of the erythrocytes (Figure 3B) in the form of teardrop-like cells (Tr) with pointed apices, sickle cells (Sk), elongated crescent-shaped RBCs, and cells with eccentric nuclei (deviating or departing from the center of the cell). The alterations of RBCs in fish exposed to MPs at 10 mg/L (Figures 3C,D) included Tr, sickle cells, eccentric nuclei, filamented cells, acanthocytes (Ac), spiculated red cells with a few projections of varying sizes and surface distributions, kidney-shaped cells (Kn), elliptocytes (slightly oval to cigar-shaped with blunt ends), and crenated cells (Cr), spherical red cells covered with short and pointed projections. In addition, the alterations of RBCs in the fish exposed to MPs at 100 mg/L (Figures 3E,F) included eccentric nuclei, filamented cells, sickle cells, Cr, Kn, elliptocytes, Ac, heart-shaped cells, and schistocytes (fragmented RBCs).

**Table 1:** Percentage of poikilocytosis cells (altered erythrocytes) of juvenile Nile tilapia (*O. niloticus)* after exposure for 15 days to microplastics (MPs) and recovery for 15 days.

	**Exposure period**				**Recovery period**			
	**Control**	**1 mg/L of MPs**	**10 mg/L of MPs**	**100 mg/L of MPs**	**Control**	**1 mg/L of MPs**	**10 mg/L of MPs**	**100 mg/L of MPs**
Hemolyzed cell	0.7 ± 0.2^a^	1.7 ± 0.3^a^	6.7 ± 0.3^b^	21.7 ± 0.6^c^	0.3 ± 0.2^a^	2.2 ± 0.3^b^	5 ± 0.4^c^	12.8 ± 0.5^d^
Sickle cell	1.2 ± 0.2^a^	3.8 ± 0.6^b^	6.8 ± 0.5^c^	15.5 ± 0.4^d^	0 ± 0^a^	3 ± 0.4^b^	3.8 ± 0.4^b^	10.2 ± 0.5^c^
Odd shapes	0 ± 0^a^	4.3 ± 0.5^b^	9.3 ± 0.7^c^	23.2 ± 0.7^d^	0 ± 0^a^	2.8 ± 0.3^b^	8.4 ± 0.4^c^	14.7 ± 0.3^d^
Schistocyte	0.7 ± 0.2^a^	4.5 ± 0.4^b^	6.5 ± 0.4^c^	20.2 ± 0.7^d^	0.7 ± 0.2^a^	4 ± 0.6^b^	6.6 ± 0.5^c^	13.3 ± 0.5^d^
Acanthocyte	1.2 ± 0.2^a^	5.4 ± 1.0^b^	10.8 ± 0.5^c^	19.2 ± 0.7^d^	0.7 ± 0.2^a^	5 ± 0.3^b^	8.8 ± 0.4^c^	16.7 ± 0.6^d^
Rouleaux	0.2 ± 0.17^a^	2 ± 0.5^b^	1.7 ± 0.3^b^	6.3 ± 0.3^c^	0 ± 0^a^	1 ± 0.3^b^	1.6 ± 0.2^b^	4.1 ± 0.3^c^
Teardrop shape	1.2 ± 0.2^a^	5.3 ± 0.95^b^	8.8 ± 0.5^c^	19.2 ± 0.5^d^	0.8 ± 0.3^a^	4.3 ± 0.4^b^	6.4 ± 0.5^c^	14.2 ± 0.3^d^
Filamented	0.5 ± 0.3^a^	4.3 ± 0.6^b^	4.3 ± 0.5^b^	10.3 ± 0.7^c^	0.5 ± 0.2^a^	3.3 ± 0.5^b^	6 ± 0.3^c^	7.5 ± 0.4^d^
Heinz bodies	0.2 ± 0.2^a^	3.3 ± 0.5^b^	6.2 ± 0.5^c^	10.7 ± 0.4^d^	0.5 ± 0.2^a^	1.8 ± 0.3^b^	3.8 ± 0.4^c^	5.3 ± 0.4^d^
Elliptocyte	1.3 ± 0.2^a^	3.3 ± 0.3^b^	6.2 ± 0.4^c^	19.8 ± 0.8^d^	0.7 ± 0.3^a^	3.5 ± 0.4^b^	7 ± 0.3^c^	9.5 ± 0.4^d^
Heart shaped	0 ± 0^a^	0.5 ± 0.2^a^	4.8 ± 0.9^b^	13.5 ± 0.4^c^	0.5 ± 0.2^a^	0.8 ± 0.2^a^	2 ± 0.3^b^	8.3 ± 0.6^c^
Eccentric nuclei	1.2 ± 0.2^a^	5.2 ± 0.4^b^	9 ± 0.6^c^	12.7 ± 0.3^d^	0.7 ± 0.3^a^	2 ± 0.3^b^	6.2 ± 0.4^c^	10 ± 0.6^d^
Crenated cell	1 ± 0.4^a^	5 ± 0.4^b^	16.2 ± 0.9^c^	21 ± 0.6^d^	0.8 ± 0.2^a^	3.7 ± 0.3^b^	9.4 ± 0.5^c^	17 ± 0.4^d^
Kidney shaped	0.7 ± 0.2^a^	2.8 ± 0.6^b^	4 ± 0.5^b^	13 ± 0.5^c^	0.2 ± 0.2^a^	1.3 ± 0.2^b^	3.8 ± 0.4^c^	10 ± 0.6^d^
Red cell agglutinate	0.3 ± 0.2^a^	0.3 ± 0.2^a^	1.7 ± 0.3^b^	9.3 ± 0.4^c^	0 ± 0^a^	0.2 ± 0.2^a^	2.2 ± 0.4^b^	2.8 ± 0.3^b^
Microcytic	1.3 ± 0.2^a^	4.8 ± 0.5^b^	10.3 ± 0.5^c^	18.3 ± 0.5^d^	0.3 ± 0.2^a^	1.7 ± 0.2^b^	7.4 ± 0.5^c^	13.2 ± 0.5^d^
Macrocytic	0.3 ± 0.2^a^	0.8 ± 0.3^a^	3.2 ± 0.4^b^	12.3 ± 0.4^c^	0.2 ± 0.2^a^	0.3 ± 0.2^a^	1.8 ± 0.3^b^	6.5 ± 0.4^c^

*Data are represented as means ± SE. The values with different superscripted letters during the exposure period in the same row for each parameter are significantly different (P < 0.05) (n = 6)*.

*The values with different superscripted letters during the recovery period in the same row for each parameter are significantly different (P < 0.05) (n = 6)*.

**Table 2:** Percentage of nuclear abnormalities of red blood cells (RBCs) of juvenile Nile tilapia (*O. niloticus)* after exposure for 15 days to MPs and recovery for 15 days.

	**Exposure period**				**Recovery period**			
	**Control**	**1 mg/L of MPs**	**10 mg/L of MPs**	**100 mg/L of MPs**	**Control**	**1 mg/L of MPs**	**10 mg/L of MPs**	**100 mg/L of MPs**
Micronuclei	1.3 ± 0.2^a^	5.8 ± 0.3^b^	9.7 ± 0.4^c^	15.2 ± 0.4^d^	1.7 ± 0.2^a^	4.7 ± 0.3^b^	6.8 ± 0.3^c^	9.7 ± 0.3^d^
Binucleated	1 ± 0.3^a^	2.3 ± 0.3^b^	4.8 ± 0.5^c^	12.3 ± 0.5^d^	0.7 ± 0.2^a^	2 ± 0.4^b^	3.8 ± 0.3^c^	7.2 ± 0.3^d^
Blebbed nuclei	0.3 ± 0.2^a^	1.8 ± 0.3^b^	4.2 ± 0.3^c^	8 ± 0.4^d^	0.3 ± 0.2^a^	0.8 ± 0.3^a^	2.3 ± 0.3^b^	5.2 ± 0.3^c^
Notched nuclei	0.3 ± 0.2^a^	2.2 ± 0.5^b^	6.2 ± 0.5^c^	13.2 ± 0.3^d^	0.3 ± 0.2^a^	1.5 ± 0.2^b^	2.3 ± 0.2^c^	6 ± 0.5^d^
Lobed nuclei	0.3 ± 0.2^a^	1.8 ± 0.3^b^	3.8 ± 0.3^c^	8 ± 0.4^d^	0.3 ± 0.2^a^	1.3 ± 0.3^b^	2.5 ± 0.2^c^	4.2 ± 0.3^d^
Hemolyzed nuclei	1.2 ± 0.2^a^	2. ± 0.3^a^	5.3 ± 0.4^b^	13.2 ± 0.6^c^	0.7 ± 0.2^a^	1.7 ± 0.2^b^	3.3 ± 0.3^c^	8 ± 0.4^d^

*Data are represented as means ± SE. The values with different superscripted letters during the exposure period in the same row for each parameter are significantly different (P < 0.05) (n = 6)*.

*The values with different superscripted letters during the recovery period in the same row for each parameter are significantly different (P < 0.05) (n = 6)*.

**Figure 4:**
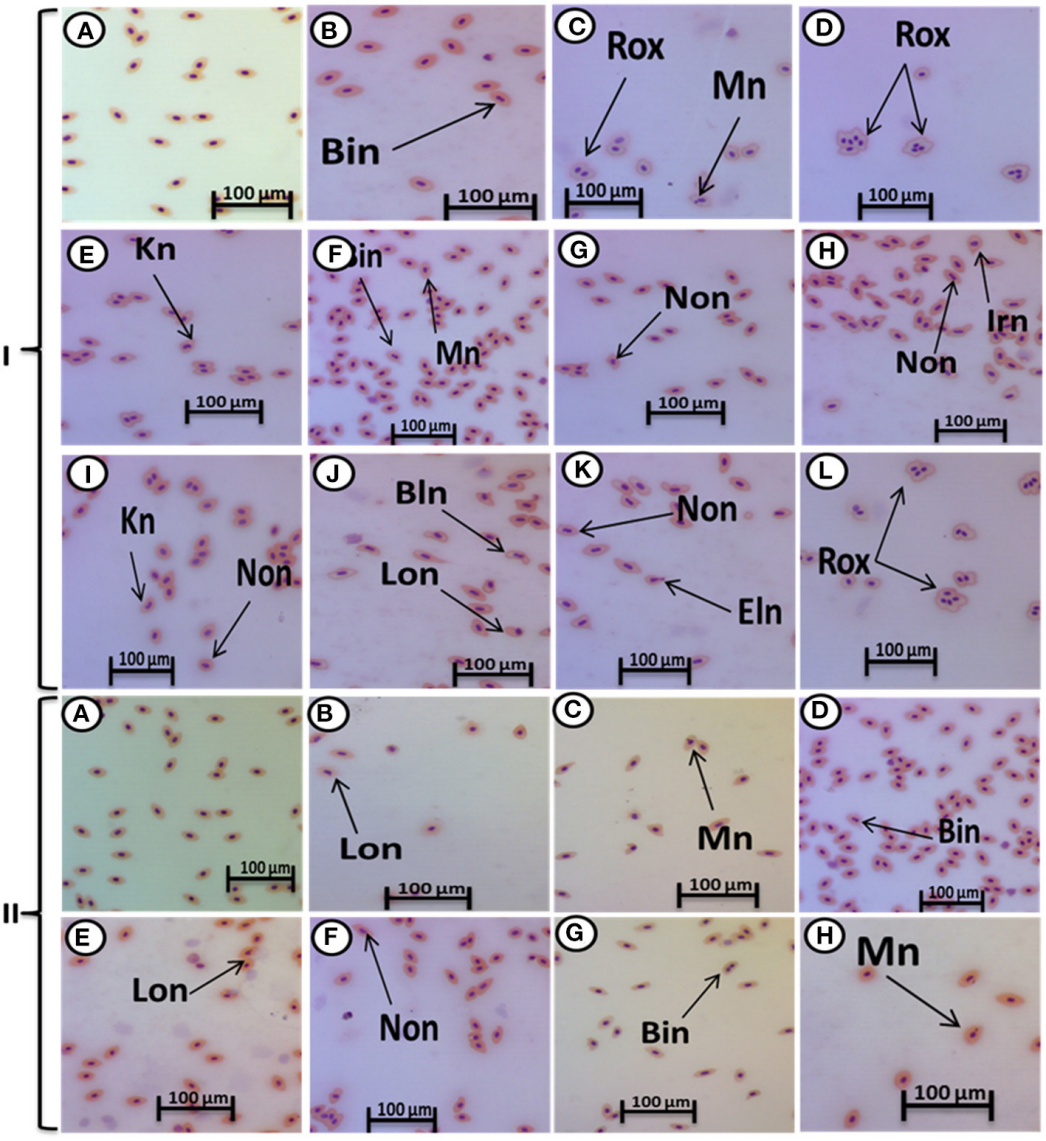
Hematoxylin and eosin-stained blood smears from juvenile (*O. niloticus)* showing nuclear abnormalities, I; MP exposure: normal erythrocytes **(A)**, deformed ones after exposure to MPs at 1 mg/L for 15 days **(B,C)**, deformed ones after exposure to MPs at 10 mg/L for 15 days **(D–G)**, and deformed ones after exposure to MPs at 100 mg/L for 15 days **(H–L)** and II; recovery period: normal erythrocytes after recovery from exposure to MPs for 15 days **(A)**, the deformed ones after recovery from exposure to MPs at 1 mg/L for 15 days **(B)**, deformed ones after recovery from exposure to MPs at 10 mg/L for 15 days **(C–E)**, and deformed ones after recovery from exposure to MPs at 100 mg/L for 15 days **(F–H)**. Bn, blebbed nucleus; En, elongated nucleus; Non, notched nucleus; Mn, micronucleus; Bin, bionuclei; Kn, kidney-shaped nucleus; Irn, irregular nucleus; Lon, lobed nucelus; Rox, rouleaux shape; Eln, elongated nucleus.

The major nuclear abnormalities of RBCs in fish exposed to MPs at 1 mg/L (Figures 4B,C) showed micronuclei (Mn), a round cytoplasmic inclusion with a diameter one-tenth to one-third that of the primary nucleus, and binuclei (Bin). The alterations of the nuclei of RBCs in fish exposed to MPs at 10 mg/L (Figures 4D–G) included Mn, Bin, Kn, notched Nuclei (Non), and the cells with clear slits that extend well into the nuclear envelope. Moreover, the alterations of nuclei of RBCs in the fish exposed to MPs at 100 mg/L (Figures 4H–L) included Non, irregular nuclei (Irn), elongated nuclei (En), Kn, Bin, lobed nuclei (Lon), and large evaginations of the nuclear envelope with no clear shape or definition. After the recovery period, a higher percentage of poikilocytosis and nuclear abnormalities of RBCs were found in the MP-treated groups compared with the control group in a concentration-dependent manner (Tables 1, 2 and Figures 3G–J, 4A–H).

## Discussion

Microplastics influenced a significant surge in apoptotic cells percentages, which is in accordance with a report by Gökçe et al. (2018), who observed that polyvinyl chloride (PVC) MPs induced apoptosis in zebrafish (*Danio rerio*) embryos in a dose-dependent manner. Moreover, Xu et al. (2019) reported that the two types of polystyrene nanoparticles (PS-NP25 and PS-NP70) caused an immense increase in apoptosis in human lung epithelial cells. Qu et al. (2019) found that the amino-modified nano polystyrene enhanced the stimulation of apoptosis compared with pristine nano polystyrene in germlines of the nematode *Caenorhabditis elegans*. Sökmen et al. (2020) reported that 20 nm polystyrene plastics can bioaccumulate brain tissue and cause apoptosis in zebrafish embryos.

Several studies reported the existence of mechanisms for apoptosis induction in cells, but details of these mechanisms are not clear. For example, Yirong et al. (2020) found that di (2-ethylhexyl) phthalate can induce apoptosis by stimulating an oxidative burst of neutrophils in carp (*Cyprinus carpio*). This apoptosis was explained by Walpitagama et al. (2019), who supported the assumptions of reactive oxygen species (ROS)–prompted apoptosis demonstrated in our previous study (Hamed et al., 2020). In addition, polystyrene upregulated the expression levels of pro-apoptotic proteins, which are strictly linked to the commencement of cell apoptosis (Xu et al., 2019; Yirong et al., 2020). In the present study, we observed that most of the characteristics of apoptosis reported in the previous studies, such as RBC shrinkage, nuclear and DNA damage, and cell-membrane alteration in erythrocytes (Lang et al., 1998; Bortner and Cidlowski, 1999, 2004; Maeno et al., 2000; Okada et al., 2001; Yu et al., 2001; Javadov and Karmazyn, 2007). These results indicate that the mechanism responsible for apoptosis in erythrocytes may involve alterations of cell components, especially ion channels (Föller et al., 2008). On the other hand, no significant differences were evident between PVC and Mater-Bi micro-debris–exposed groups and control groups concerning apoptosis in *Dreissena polymorpha* (Magni et al., 2020).

In this study, MPs influenced an immense surge in the percentage and nuclear abnormalities of poikilocytosis cells in RBCs compared with the control group. To the best of our knowledge, this is the first study to illustrate the effect of MPs on the poikilocytosis of RBCs. We concluded that MPs or their additives interact with erythrocytes and limit the dehydrogenase of delta-aminolevulinic acid and induce the interruption of plasma membranes, producing abnormally shaped blood cells (poikilocytosis) (Hamed et al., 2019b). The higher production of ROS in erythrocytes could offer a reasonable explanation for this phenomenon; it could be caused by the direct interaction between MPs and erythrocyte plasma membranes (da Costa Araújo et al., 2020a).

In our results, the nuclear abnormalities of RBCs included Mn, binucleated erythrocytes, notched, lobed, blebbed, and hemolyzed nuclei. A higher percentage of nuclear abnormalities (and Mn in particular) were observed in *Mytilus galloprovincialis* mussels after exposure to the polyethylene and polystyrene MPs (Avio et al., 2015) and benzo(a)pyrene and/or benzo(a)pyrene-contaminated low-density polyethylene (Pittura et al., 2018). Polyethylene MPs induced nuclear anomalies, e.g., Mn, NoN, blebbed, binucleated or multilobed nuclei, kidney-shaped nuclei, eccentric nuclei, and nuclear vacuoles in erythrocytes of *Danio rerio* after feeding on fry of *Poecilia reticulate* as well as in erythrocytes of *Physalaemus cuvieri* tadpoles (da Costa Araújo et al., 2020b).

The nuclear anomalies may be a product of aneuploidy or disruption of cytokinesis (da Costa Araújo et al., 2020a) as well as the elimination of amplified genes (Crott et al., 2001). Strunjak-Perovic et al. (2009) reported that the nuclear anomalies may result from gene mutations in the nuclear lamina of the nucleus. Moreover, da Costa Araújo et al. (2020a) found that the genotoxicity of polyethylene MPs on erythrocytes was indirectly induced by the formation of free radicals that interfere with DNA integrity, and our previous study linking oxidative stress in tilapia with exposure to MPs (Hamed et al., 2020) reinforces this conclusion. On the contrary, no significant differences between PVC and Mater-Bi micro-debris–exposed groups and the control group were found in *Dreissena polymorpha* Mn (Magni et al., 2020).

After the recovery period, a higher percentage of apoptotic cells and poikilocytosis and nuclear abnormalities of RBCs were still observed in the MP-treated groups in a dose-dependent manner. This could be attributed to the fact that apoptosis is generally believed to be irreversible (Tang et al., 2012).

In addition, Gagnaire et al. (2013) showed that the depleted uranium surged ROS production in adults and larvae even at the low concentrations and the depuration period for adult *D. rerio*. In addition, Stankevičiute et al. (2016) found that Mn incidences were found to immensely surge after a 4-day recovery in all the tissues. Blood erythrocytes maintained noticeable recovery of all analyzed geno-cytotoxicity endpoints after 8 and 12 days in the 0.25 group; however, the recovery was observed after 12 days of depuration in the liver and kidney erythrocytes. Martins et al. (2018) relayed that the inflammation and formation of hyperplastic foci in fish epithelia showed slower recovery and unexpected increased expression of a *ras* family oncogene homolog following depuration. Removing the chemical intruders could enhance tissue recovery, though it does not completely clear the molecular and histopathological endpoints commonly related to neoplasia. Détrée and Gallardo-Escárate (2018) stated that during the period of recovery, a contrasting response was found with the activation of apoptotic processes and the upregulation of immune receptors and stress-related proteins (glutathione peroxidase, hsp70) in mussels previously exposed to MPs. This states that the physiological stress and physical damages induced by MPs persist after an event of exposure. A 30-day depuration period induces a tendency to recover initial values of biomarkers determinations in the fish liver of sea bream after long-term exposure to virgin and seawater affected by a MPs enriched–diet. Yet, it seems to suffice for its complete normalization (Capó et al., 2021).

In contrast, Solomando et al. (2021) surmised that the liver and blood biomarkers of *Sparus aurata* managed to recover during the period of depuration, with the majority reaching levels close to those of the control group after long-term exposure to MPs. In addition, Iheanacho and Odo (2020) observed that *Clarias gariepinus* fed a diet containing PVC particles (0, 0.5, 1.5, and 3.0%) for 30 days, presented a reduction in erythrocyte mean cell volume, neutrophil counts, cell hemoglobin, and induced oxidative stress in brain and gills. For 45 days, a depuration period allowed the recovery of most of the analyzed parameters.

## Conclusion

The fish RBCs can be sensitive and reliable biomarkers for the measurement of the cytotoxic and genotoxic effects of MPs. Exposure of tilapia to MPs induces apoptosis, poikilocytosis, and nuclear abnormalities of RBCs. Following the period of recovery, almost all the detected variances were still evident in the MP-treated groups in a dose-dependent manner. This could be attributed to the fact that these changes are generally believed to be irreversible or the necessity of a longer recovery period for the fish.

## Data Availability Statement

The raw data supporting the conclusions of this article will be made available by the authors, without undue reservation.

## Ethics Statement

The animal study was reviewed and approved by Assiut University. Experimental setup and fish handling were approved within the research by the Ethical Committee of the Faculty of Science, Al-Azhar University, Assiut Branch, Egypt. This study does not contain human or plant subjects, and all applicable international, national, and/or institutional guidelines for the care and use of animals were adhered to.

## Author Contributions

MH, AO, HS, and AE-DS: experimental design. MH, HS, and AE-DS: data interpretation. MH: Biochemical analysis. MH, AO, AB, HS, and AE-DS: writing and revision. All authors have read and approved the final manuscript.

## Conflict of Interest

The authors declare that the research was conducted in the absence of any commercial or financial relationships that could be construed as a potential conflict of interest.

## Publisher's Note

All claims expressed in this article are solely those of the authors and do not necessarily represent those of their affiliated organizations, or those of the publisher, the editors and the reviewers. Any product that may be evaluated in this article, or claim that may be made by its manufacturer, is not guaranteed or endorsed by the publisher.
